# m6A-Related Genes Contribute to Poor Prognosis of Hepatocellular Carcinoma

**DOI:** 10.1155/2022/2427987

**Published:** 2022-10-26

**Authors:** Yan Zou, Guichun Jiang, Yanmin Xie, Hui Li

**Affiliations:** ^1^Endoscopy Center, Liaoning Province Cancer Hospital & Institute (Cancer Hospital of China Medical University), No. 44 Xiaoheyan Road, Dadong District, Shenyang City, Liaoning Province, China 110042; ^2^Liaoning Province Cancer Hospital & Institute (Cancer Hospital of China Medical University), No. 44 Xiaoheyan Road, Dadong District, Shenyang City, Liaoning Province, China 110042

## Abstract

**Background:**

Hepatocellular carcinoma (HCC) is one of the most common and lethal digestive system cancers worldwide. N6-methyladenosine (m6A) modification plays an essential role in diverse critical biological processes and may participate in the development and progression of HCC.

**Methods:**

We downloaded transcriptome data and clinical data from TCGA as the training set. COX and LASSO screened prognostic m6A genes. ROC and Kaplan-Meier curve analysis evaluated the effectiveness of the model. ICGC and our center data were used as verification sets.

**Results:**

We include the “writer (METTL3, METTL14, WTAP, KIAA1429, RBM15, ZC3H13),” the “reader (YTHDC1, YTHDC2, YTHDF1, YTHDF2, HNRNPC),” and the “eraser (FTO, ALKBH5)” in the study. We obtained YTHDF2, YTHDF1, METTL3, and KIAA1429 through differential analysis, survival analysis, and LASSO regression analysis. The prediction model was established based on the expression of these 4 molecules. HCC patients were divided into “high-risk” and “low-risk” groups to compare survival differences. The model suggested a poor prognosis in the validation sets.

**Conclusion:**

The four-m6A-related-gene combination model was an independent prognostic factor of HCC and could improve the prediction of the prognosis of HCC.

## 1. Introduction

Hepatocellular carcinoma (HCC) is a common human malignant tumor of the digestive system, and its pathogenesis is related to many factors, such as aflatoxin infection, hepatitis B virus infection, and excessive alcohol consumption [1]. According to the latest data, there is still more than one million new HCC worldwide yearly. Although the incidence and mortality of HCC have declined globally in the past five years, HCC is still the third leading cause of cancer-related deaths [2]. In recent years, magnetic resonance imaging (MRI) has been popularized in many countries; the early diagnosis of HCC has been improved to a certain extent, but because early HCC often has no obvious clinical symptoms, most HCC is already in the advanced stage at the time of diagnosis, and the patient population is gradually younger. Although significant progress has been achieved in treating HCC, the prognosis of HCC patients remains unsatisfactory [3]. According to research, the 5-year survival rate of advanced HCC in China is less than 30%. Thus, HCC diagnosis and treatment still face a considerable challenge. It is extremely urgent to deepen the understanding of the pathogenesis of HCC development and seek novel biological markers or a therapeutic target [4].

Epigenetics refers to the fact that the genome interacts with the environment to influence gene expression and carry out stable inheritance without affecting the DNA sequence [5]. It mainly includes DNA methylation, histone modification, chromatin remodeling, and RNA modification. At present, we have a more thorough understanding of the epigenetic modification related to DNA methylation and histone modification, but the methylation modification of RNA is still a relatively unknown area [6]. N6-methyladenosine (m6A) refers to the methylation modification of the sixth nitrogen atom of adenine, which is the most common mRNA methylation modification in mammals [7]. The m6A modification was first discovered in the 1970s, but only in recent years, with the development of proteomics and high-throughput detection technology, has m6A modification been associated with disease development and cellular processes. Its abnormal regulation is considered to be an essential factor in tumorigenesis and development [8].

Many factors are involved in the dynamic regulation of m6A methylation modification of mRNA. “Writers,” METTL3 (methyltransferase-like 3), or METTL14 (methyltransferase-like 14) catalyze the modification of m6A on RNA by forming a methyltransferase complex [9, 10]. “Eraser,” FTO (alpha-ketoglutarate-dependent dioxygenase), and ALKBH5 (alkylation repair homologous protein 5) remove the m6A modification of mRNA [11, 12]. The “reader” can recognize m6A modifications and regulate mRNA function and metabolism. The YTHDF protein family is the first confirmed reader protein. YTHDF1 can recognize the m6A modification of mRNA and accelerate the translation process [13, 14]. After YTHDF2 recognizes the m6A modification, it promotes mRNA processing and transfer to the cytoplasm, accelerates mRNA degradation, and inhibits protein translation [15]. It suggested that the abnormal expression of m6A-related proteins is related to tumor occurrence and development [16]. The unregulated expression of m6A-related genes may indicate the poor prognosis of HCC.

In this study, we obtained HCC transcriptome and clinical data from The Cancer Genome Atlas (TCGA) as a training set. Based on expression analysis, Cox regression, and least absolute shrinkage selection operator (LASSO) analysis, a multi-m6A-related gene combination survival prediction model was constructed. The International Cancer Genome Consortium (ICGC) data and our center's data were used as the internal verification set and the external verification set to verify the clinical validity of the model. This model may help evaluate the prognosis of HCC patients.

## 2. Methods

### 2.1. Data Download

We downloaded RNA sequencing data and corresponding clinical data of HCC from TCGA and ICGC databases as the training set and internal validation set, respectively. There were 374 HCC and 50 normal samples in the TCGA database for bioinformatics analysis. R and Phyton were used to organize data.

### 2.2. Data Analyses

We extracted the expression matrix of m6A-related genes from the expression profile data (Sup Table 1). Package “limma” was used for difference analysis, and *P* < 0.05 and fold change ≥ 2 were set as the cutoff values. Clinical information was displayed in Sup Table 2.

The R package “pheatmap” and “vioplot” were applied to visualize the expression of m6A-related genes. The Search Tool for the Retrieval of Interacting Genes (STRING, http://string.embl.de/) and R package “corrplot” were used to analyze and show the correlation between these genes. The R prcomp was used to perform principle component analysis (PCA) using the RNA-seq data. Clustering analysis was performed with the Seurat R package v3.1.5. The univariate and multivariate survival analyses utilized the “survival” package. Differentially expressed and survival-related genes were included in the LASSO analysis to establish a regression model (package “glmnet”). It was visualized by the package “survminer.” The next grouping was based on gene expression, and the median was set as the cutoff value. Visualization was performed with ggplot2 packages.

### 2.3. Cell Culture and Tissue Samples

Hepatocellular cell lines (THLE-3) and human HCC cell lines (Huh7 and Hep3B) were acquired from the Central Laboratory of Liaoning Cancer Hospital (Shenyang, China). RPMI-1640 medium (Gibco, Carlsbad, CA, USA) contained 10% fetal bovine serum- (FBS-) implemented cell culture at 37°C with 5% CO_2_.

One hundred ten pathologically diagnosed HCC samples and matched nontumor tissue samples were included in the study. All samples are from HCC patients who underwent surgical resection in Liaoning Cancer Hospital from 2010 to 2015. All patients signed an informed consent form before surgery, and the Ethics Committee approved the study of Liaoning Provincial Cancer Hospital & Institute (IRB number: 20181215). The end date of follow-up was 2021-08-31. The patients' characteristics are described in Sup Table 3.

### 2.4. Reverse Transcription-Polymerase Chain Reaction (RT-PCR)

The TRIzol method was used to extract total RNA from tissues and cells. A UV spectrophotometer detected the concentration and purity of RNA. When A260/A280 = 1.8 − 2.0, the concentration and purity of expressed RNA were qualified, and the next step could be carried out. By reverse transcription, mRNA was synthesized into cDNA and stored in a refrigerator at -80°C. Subsequently, qRT-PCR could be performed. RT-PCR system is as follows: 10x buffer 2.5 *μ*L, cDNA 1 *μ*L, forward primer (20 *μ*mol/L) 0.5 *μ*L, and reverse primer (20 *μ*mol/L) 0.5 *μ*L. LightCycler® 480 SYBR Green I Master (2x): 10 *μ*L; ddH2O: 5.5 *μ*L. The sequence information is listed in Sup Table 4.

### 2.5. Statistical Analyses

All the data were analyzed by SPSS 22.0 software (IBM, Armonk, NY, USA). The quantitative data derived from three independent experiments were expressed as mean ± standard deviation (SD). Statistical tests were performed using Student's *t*-test or one-way ANOVA with Tukey's post hoc test. Disease-free survival (DFS) and overall survival (OS) were calculated using the Kaplan-Meier method, and the log-rank test was used to compare Kaplan-Meier curves. LASSO regression analysis was performed to reduce overfitting caused by univariate. Hazard ratios (HR) were estimated with a Cox proportional hazards model. *P* < 0.05 was accepted as statistically significant.

## 3. Results

### 3.1. The Expression of m6A-Methylated Genes in HCC

**Figure 1:**
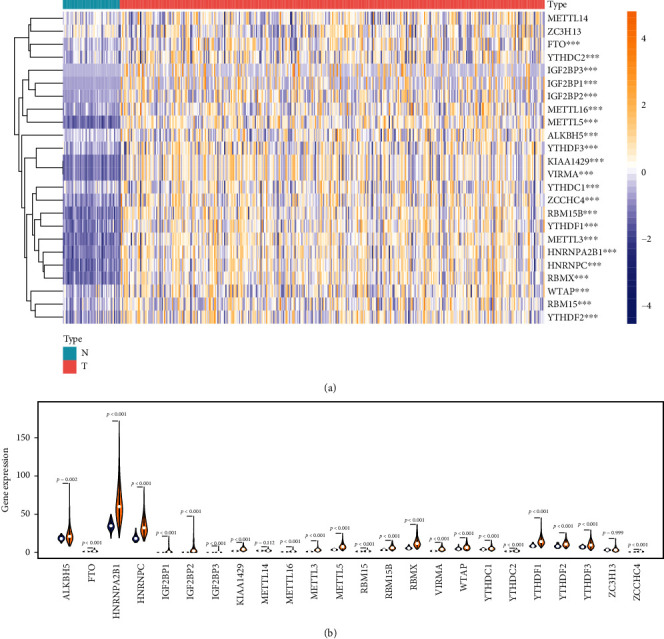
The expression of m6A methylation genes in HCC. (a) Heat map showed the expression of the m6A methylation genes in 374 HCC and 50 adjacent tissues. (b) Vioplot visualized the expression of 13 m6A methylation genes in different tissue samples in HCC and adjacent tissues.

Based on existing studies, this research included a total of 24 m6A-related genes as the research object. 21 of them are from the m6A2Target database (http://m6a2target.canceromics.org/) [17], which is the most commonly used m6A database in bioinformatics analysis. Besides, it also includes 3 other genes that have been shown to be associated with m6A, but relatively few have been reported (Figure 1(a)). We downloaded 424 HCC-related transcriptome data from TCGA. Among them, there were 374 HCC tissues and 50 adjacent tissues. *P* < 0.05 and |fold change| ≥ 2 were set as the cutoff values to evaluate the expression of the above genes by package “limma.” Ten genes were abnormally expressed (Figures 1(a) and 1(b)).

### 3.2. The Correlation of m6A Methylation Gene Expression in HCC

**Figure 2:**
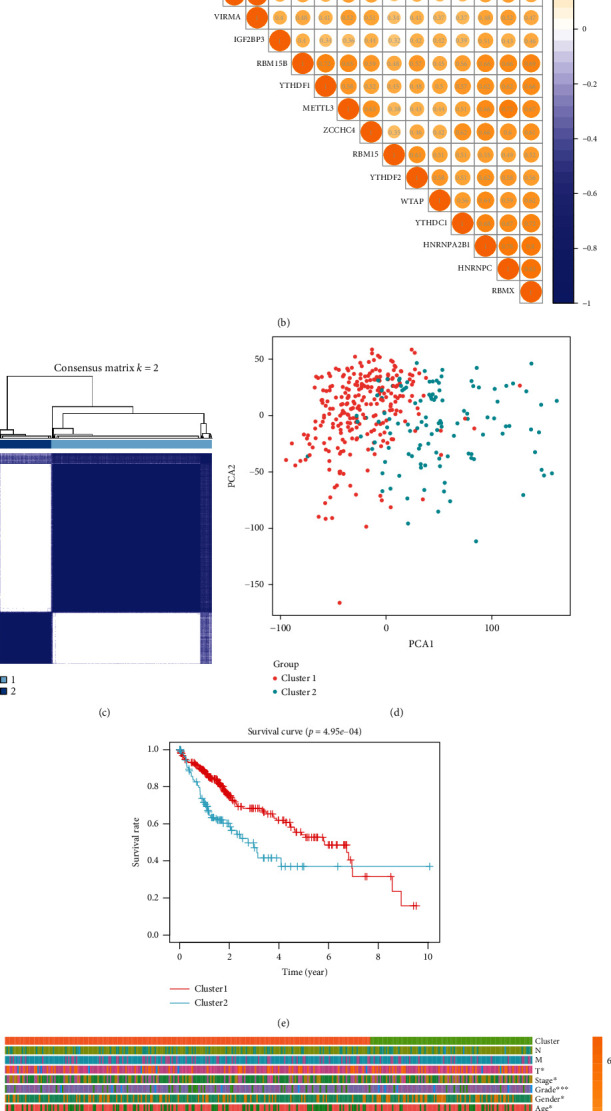
Identification of coexpressed gene clusters of m6A methylation genes. (a) m6A methylation gene interaction network constructed by STRING database. (b) Spearman analysis of the correlation of m6A methylation genes in HCC. (c) Methylated genes could be clustered into two consistency matrices. (d) Principal component analysis showed that these two clusters could distinguish HCC patients well. (e) The Kaplan-Meier curve was used to analyze the overall survival of the two subgroups. (f) The heat map showed the correlation between the two subgroups and clinicopathological data.

Subsequently, we further analyzed the relationship among these m6A-related genes. We obtained the protein-protein interaction (PPI) network from the STRING database (https://string-db.org). Not surprisingly, there was an evident and complex interaction among m6A-related genes (Figure 2(a)). Subsequently, we analyzed the correlation of these gene expressions. Among them, “writer” METTL3 and “reader” HNRNPC had the strongest correlation. Whether it suggested that HNRNPC had a better recognition effect on the methylation modification that METTL3 participated in was still unknown (Figure 2(b)). Cluster analysis showed that m6A-related genes could be appropriately divided into two clusters (Figure 2(c)). Principal component analysis (PCA) showed that molecular clusters could easily distinguish cancer and paracancerous tissues (Figure 2(d)). Furthermore, they could identify a poor prognosis (Figure 2(e)). The molecular cluster classification was closely related to the TNM staging and tumor differentiation of HCC (Figure 2(f)). The above results suggested that the clinical characteristics of HCC could be evaluated by m6A-associated gene classification.

### 3.3. A Prediction Model Based on the Coexpression of m6A-Related Genes

**Figure 3:**
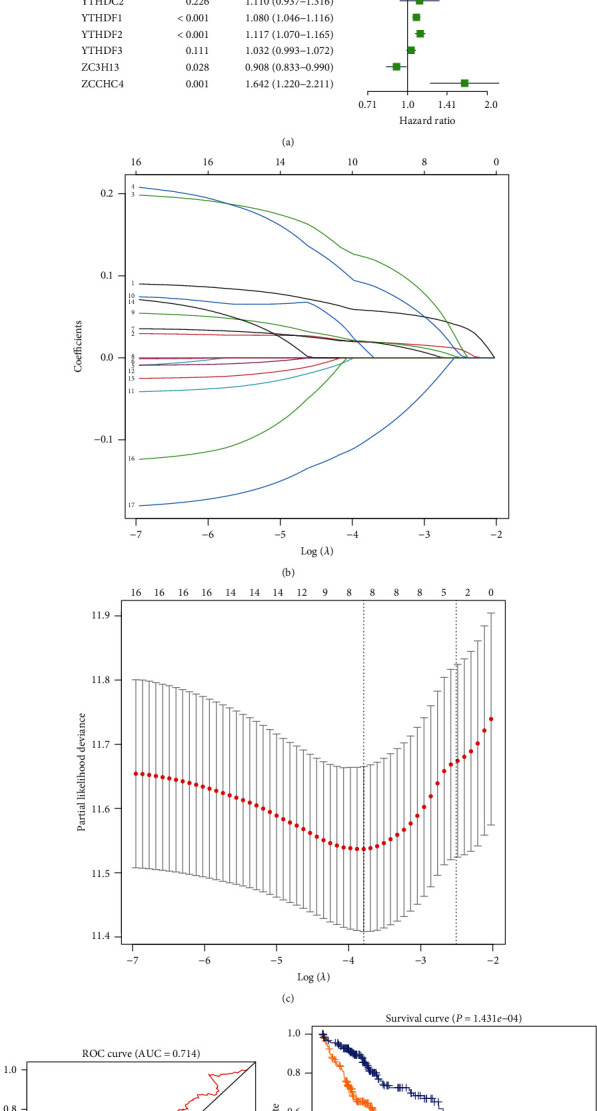
Risk signature with m6A methylation genes. (a) Univariate Cox regression calculated the hazard ratios (HR) and 95% confidence intervals (CI) of m6A methylation genes. (b) Incorporate 8 differential genes with prognostic significance into LASSO. (c) L1-penalty of LASSO-COX regression. The dotted vertical lines at optimal log (Lambda) value: 4. (d) ROC assesses predictive model validity. (e) Patients were divided into high-risk and low-risk groups based on risk scores, and survival curves were plotted.

Furthermore, we performed a univariate survival analysis on m6A-associated genes based on the clinical data in the TCGA database. The abnormal expression of YTHDF2, YTHDF1, METTL3, KIAA1429, HNRNPC, WTAP, YTHDC1, RBM15, and ZC3H13 indicated a poor prognosis (Figure 3(a)). We selected eight genes for subsequent analysis according to the results of univariate survival analysis and differential expression gene analysis. In the analysis process, we use the least absolute contraction and selection operator (LASSO) analysis to effectively avoid overfitting caused by univariate Cox regression (Figures 3(b) and 3(c)). Therefore, we obtained a four-m6A-associated gene combination prognostic model for HCC patients (risk score = YTHDF2 × 0.059 + YTHDF1 × 0.027 + METTL3 × 0.066 + KIAA1429 × 0.034, Figure 3(c)). ROC curve and C index were used to evaluate the prognostic model. ROC analysis revealed that the area under the ROC curve was 0.714, with a *P* < 0.01. The c-index was 0.717; 95%CI = 0.643 − 0.791. This model could effectively evaluate the prognosis of HCC (Figure 3(d), *P* < 1.431 × 10^−4^).

### 3.4. Validity of Prognostic Models

**Figure 4:**
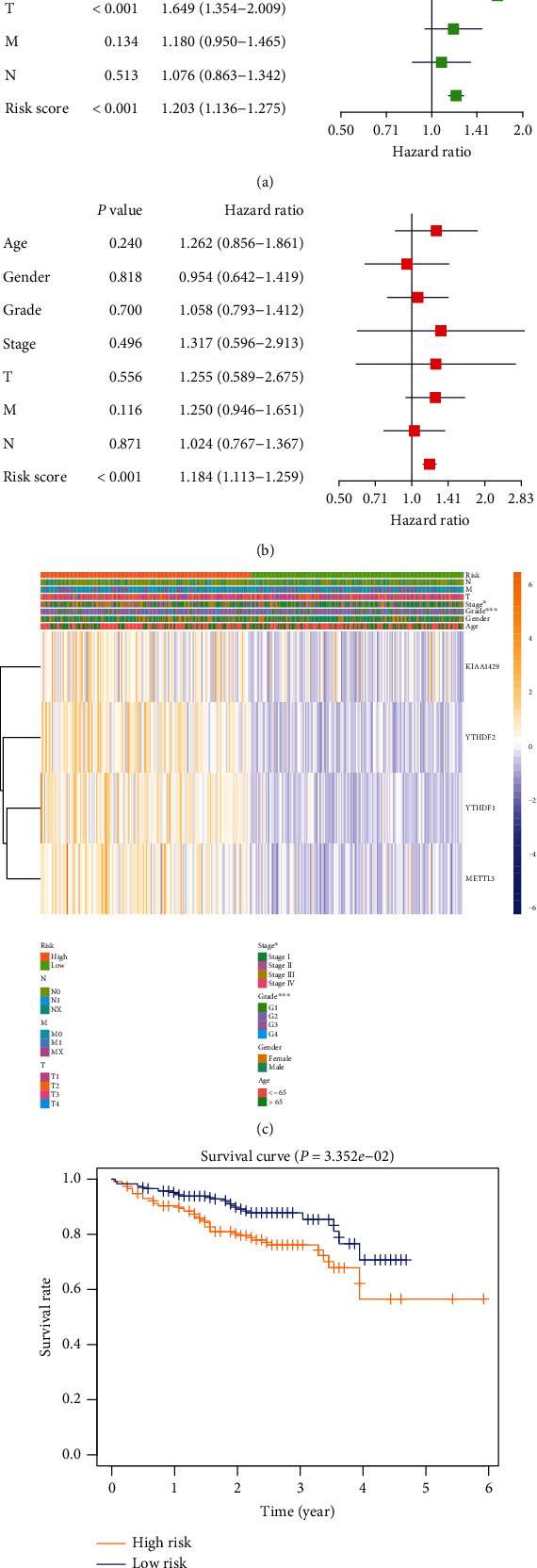
Relationship between risk prediction model and clinicopathological features and prognostic value. (a) Univariate Cox regression analysis showed that risk score, T stage, and TNM stage were poor prognostic factors. (b) Multivariate Cox regression analysis exhibited that risk score was an independent risk factor for the prognosis of HCC. (c) Heat map showed the expression of two m6A RNA methylation regulators in GC. The distribution of clinicopathological features was compared between high-risk and low-risk groups. (d) Based on the survival data of HCC in ICGC, the high-risk group suggested a poor prognosis.

In order to further evaluate the clinical significance of the model, we comprehensively analyze the model and clinicopathological factors. In univariate analysis, the TNM stage, T stage, and prognostic model were risk factors for the poor prognosis of HCC (Figure 4(a)). Furthermore, this model was the only independent prognostic risk factor in multivariate analysis (Figure 4(b)). Excitingly, the model was closely related to clinical staging (*P* < 0.05) and tumor differentiation (*P* < 0.001, Figure 4(c)). In order to evaluate the effectiveness of the modified model, we downloaded the transcription and clinical prognosis data of HCC from the ICGC database (https://dcc.icgc.org/releases/current/Projects/LIRI-JP). The training set data in this study came from TCGA's global data, and the validation set data came from Asian data in ICGC, effectively avoiding selection bias. We scored the ICGC data based on the expression of YTHDF2, YTHDF1, METTL3, and KIAA1429. The median was set as the cutoff value to group the clinical data. Obviously, the high-risk group had a poorer prognosis (Figure 4(d)). The series analyses confirmed the model's effectiveness in evaluating the prognosis of HCC.

**Figure 5:**
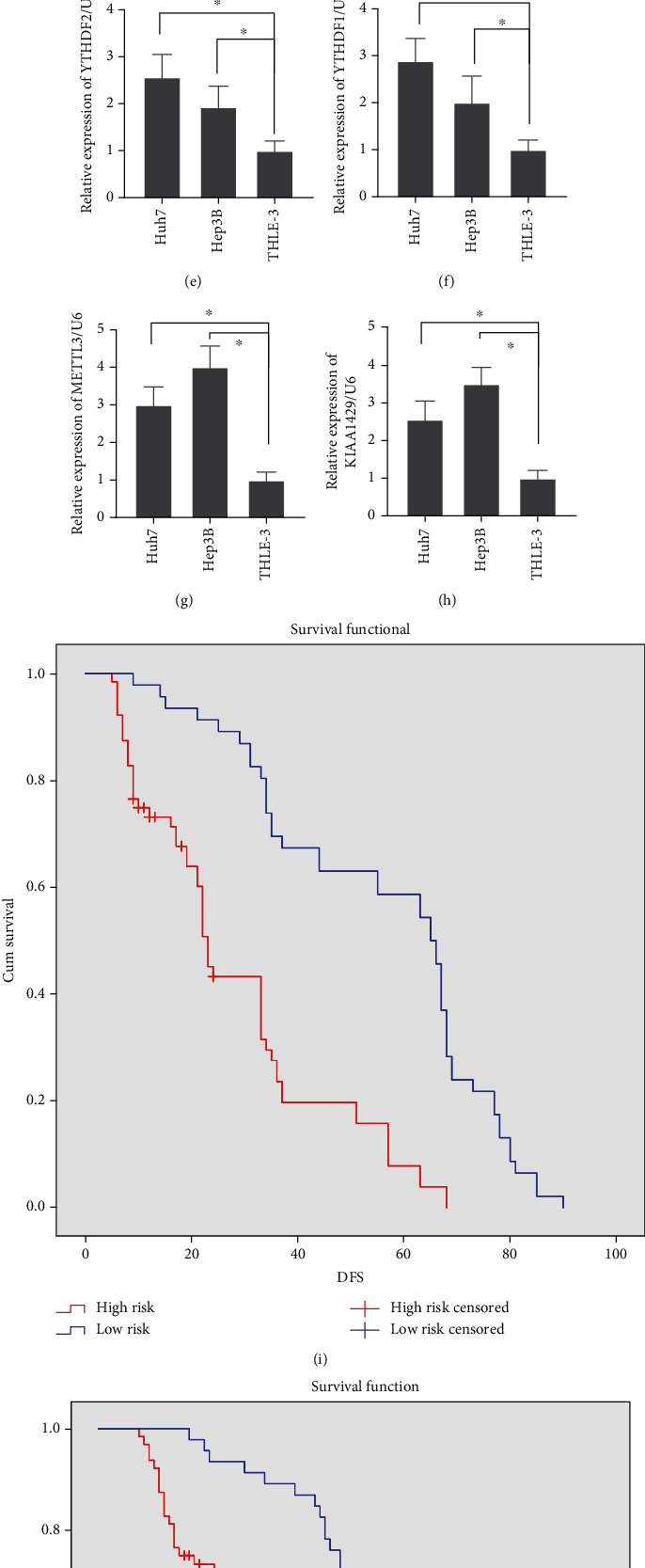
The expression of YTHDF2, YTHDF1, METTL3, and KIAA1429 in HCC cells and tissue. (a, e) YTHDF2 was overexpressed in HCC tissue and cells. (b, f) YTHDF1 was overexpressed in HCC tissue and cells. (c, g) METTL3 was overexpressed in HCC tissue and cells. (d, h) KIAA1429 was overexpressed in HCC tissue and cells. (i, j) High risk contributed to the poor DFS and OS.

Subsequently, we tested the expression of YTHDF2, YTHDF1, METTL3, and KIAA1429 in HCC cells and tissue samples. Consistent with the TCGA data, all YTHDF2, YTHDF1, METTL3, and KIAA1429 were overexpressed in HCC tissues (Figures 5(a)–5(d)). In addition, they were upregulated in HCC cells compared to normal hepatocellular cells (Figures 5(e)–5(h)). Based on their expressions, we divided 110 patients into high-risk and low-risk groups, with the median as the cutoff value. Obviously, both the DFS (disease-free survival) and OS (overall survival) of the high-risk group were shorter (Figures 5(i) and 5(j), Table 1). Moreover, this model, like tumor size, distant metastasis, and TNM stage, was also an independent risk factor for the prognosis of HCC (Table 2).

## 4. Discussion

Traditional research believed that gene mutations were the main reason for the occurrence and development of cancers, including HCC [18]. However, with the progress of “omics” research, epigenetic modification has gradually entered the field of view of researchers [19]. DNA methylation, microRNA (miRNA), long-chain noncoding RNA (lncRNA), and histone acetylation have been confirmed to be involved in tumorigenesis [19]. Scientists regarded these modifications as essential indicators of tumor treatment or for judging the prognosis of patients [20]. There were 163 different RNA chemical modifications that had been identified in all organisms as of the end of 2017. Among these modifications, m6A was considered the most common, abundant, and conservative modification in eukaryotic mRNA, miRNA, and lncRNA, which affected RNA transcription, processing, translation, and metabolism [20].

m6A was a dynamic process. Many studies focused on the role of m6A in HCC, but there were still contradictions. Ma et al. found that the m6A level in HCC tissues was downregulated, and the differences in the expression of various RNA methylases were widely recognized. The expressions of METTL3 and METTL14 were significantly downregulated in the methylase catalytic complex. The article focused on the function of METTL14 and verified that it could affect the maturation of metastasis-related miRNAs and inhibit the growth and metastasis of HCC [21]. However, a few months later, Chen et al. found that the m6A level in HCC was upregulated in the TCGA database and tissue. METTL3 was overexpressed and could silence SOCS2 through m6A-dependent pathways to promote the progress of HCC. Besides, METTL14 could also boost the progress of HCC in the Huh7 cell [22]. Similarly, another research team also found that downregulated METTL14 expression could promote HepG2 cell invasion and metastasis [23]. In addition, WTAP and KIAA1429 were highly expressed in HCC, regulated the binding of HUR to target RNA in an m6A-dependent mechanism, and promoted the progress of HCC [24, 25]. In all, different cell line selections, different HCC tissue sources, and experimental conditions might lead to the above contradictory results. However, one thing is undeniable: m6A participates in the occurrence and development of HCC with complex functions.

The YTHDF protein family was the first confirmed m6A “Reader” protein. The research on the mechanism of YTHDF2 in HCC was relatively in-depth at present. YTHDF2 participated in METTL3-mediated SOSC2 silencing to promote the progress of HCC [22]. Studies had also reported that the overexpressed YTHDF2 could inhibit the growth and metastasis of HCC and be closely related to the inflammatory environment [26, 27]. Inconsistently, it was also found that knocking down YTHDF2 significantly inhibited HCC growth and metastasis potential [28]. In addition, the other m6A associate genes, such as HNRNPA2B1, EIF3, and IGF2BPs, had been confirmed to participate in HCC by a large number of studies in the past. However, as of now, there were no reports about their involvement in the progress of HCC through the m6A mechanism [29, 30]. Therefore, it may be of great benefit to further improve the prognosis of HCC to explore the role and clinical significance of m6A-related genes.

In this study, we analyzed the expression of m6A-related methylases in HCC and discussed their relevance to clinical prognosis. Based on existing studies, we included a total of 24 m6A-related genes in the updated manuscript. 21 of them are from the m6A2Target database (http://m6a2target.canceromics.org/) [17]. This database is by far the most commonly used m6A database in bioinformatics analysis. Besides, it also includes 3 other genes that have been shown to be associated with m6A, but relatively few have been reported. We used Cox and LASSO regression to establish a risk prediction model based on YTHDF2, YTHDF1, METTL3, and KIAA1429 expression. LASSO regression can effectively reduce the overfitting caused by univariate COX regression [31, 32]. In addition, it improves the accuracy and interpretability of prediction through variable selection and regularization. The modeling process of LASSO regression includes the relationship with ridge regression, the best subset selection, and the link between the LASSO coefficient estimation and the soft threshold [33, 34]. It is an efficient regression analysis method in statistics and machine learning [35, 36]. Moreover, LASSO regression, ridge regression, and elastic net regression regularization methods maintain good results in the presence of high dimensionality and multicollinearity among the variables in the dataset [37]. LASSO regression is very similar to ridge regression, and both techniques add a bias term to the regression optimization function to reduce the effect of collinearity and thus reduce model variance. However, LASSO regression uses absolute deviations as a regularization term instead of squared deviations like ridge regression, making it computationally more efficient [38]. To a certain extent, elastic net regression adds a penalty term to the LASSO model, which is more suitable for elastic net [39], but its application in transcriptome data analysis is still lacking evidence. Currently, Pak et al. have developed online analysis tools based on these regression analyses [39], which may be helpful for further screening of molecular models.

**Figure 6:**
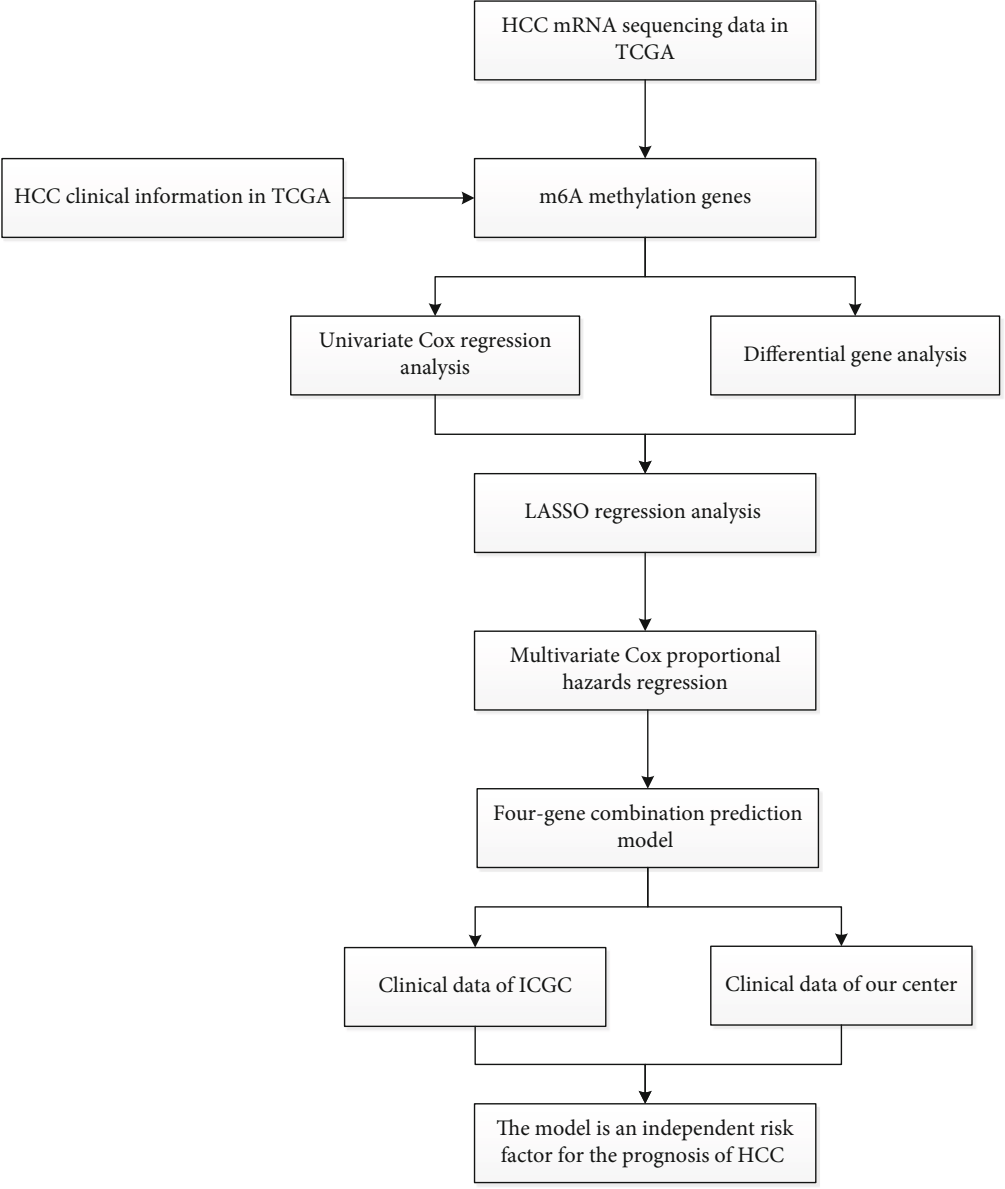
The workflow of the study.

Subsequently, we evaluated the effectiveness of this model in predicting the prognosis of HCC based on ICGC and clinical data from our center. Moreover, we got positive results. The high-risk group reflected poor survival both in ICGC and our center. It was an independent risk factor for the poor prognosis of HCC (Figure 6).

## 5. Conclusion

In all, the further application of this model may have crucial clinical significance for predicting the prognosis of HCC. It may bring new ideas for the follow-up targeted therapy of HCC. However, the mechanism of YTHDF2, YTHDF1, METTL3, KIAA1429, and others in HCC still needs to be actively explored. More m6A-methylated genes and mechanisms need to be identified.

## Figures and Tables

**Table 1:** Patient's pathological feature and univariate analysis.

Characteristics	*N*	DFS			OS		
		Month	*P* value	*χ* ^2^	Month	*P* value	*χ* ^2^
Age			0.23	1.34		0.20	1.66
≥65	63	38.55			45.64		
<65	47	43.48			48.86		
Gender			0.39	0.75		0.30	1.06
Male	60	37.56			44.79		
Female	50	44.19			49.59		
AFP (*μ*g/L)			0.56	0.33		0.42	0.66
<20	56	44.38			50.14		
≥20	54	37.65			44.50		
HbsAg			0.56	0.35		0.37	0.80
Positive	73	39.93			46.45		
Negative	37	42.02			48.11		
Cirrhosis			0.39	0.73		0.29	1.13
Present	59	37.36			44.29		
Absent	51	44.28			50.05		
Tumor size			0.01	8.13		0.01	6.78
≥5 cm	57	35.37			42.00		
<5 cm	53	45.88			51.98		
Tumor number			0.01	9.60		0.01	10.57
Multiple	49	34.46			43.00		
Solitary	61	44.38			49.28		
Vascular invasion			0.01	7.94		0.01	8.69
Yes	42	30.45			37.86		
No	68	46.80			52.58		
Capsule			0.01	11.61		0.01	12.71
Absence	47	49.15			54.83		
Presence	63	33.13			40.11		
Distant metastasis			0.01	26.81		0.01	26.70
Absence	47	26.64			34.88		
Presence	63	49.72			55.19		
TNM stage			0.01	38.60		0.01	39.88
I	12	78.64			78.64		
II	33	45.85			53.35		
III	65	31.07			38.13		
Model			0.01	37.78		0.01	41.41
High risk	64	28.09			35.69		
Low risk	46	55.76			60.76		

**Table 2:** Multivariate analysis of significant prognostic factor for survival in HCC patients.

Variables	DFS			OS		
	*P* value	HR	95% CI	*P* value	HR	95% CI
Tumor size	0.01	0.43	0.27-0.68	0.01	0.42	0.26-0.67
Tumor number	0.45	0.82	0.50-1.36	0.32	0.77	0.46-1.29
Vascular invasion	0.09	0.67	0.42-1.06	0.08	0.65	0.41-1.05
Capsule	0.10	1.46	0.93-2.29	0.07	1.52	0.96-2.40
Distant metastasis	0.01	0.27	0.16-0.46	0.01	0.26	0.15-0.44
TNM stage	0.01	3.91	2.50-6.12	0.01	4.13	2.60-6.56
Risk score	0.01	0.42	0.26-0.70	0.01	0.37	0.22-0.62

## Data Availability

No additional data are available.

## Abbreviations

HCC: Hepatocellular carcinoma
MRI: Magnetic resonance imaging
m6A: N6-methyladenosine
METTL3: Methyltransferase-like 3
METTL14: Methyltransferase-like 14
FTO: Obesity-associated protein
ALKBH5: Alkylation repair homologous protein 5
TCGA: The Cancer Genome Atlas
LASSO: Least absolute shrinkage selection operator
ICGC: The International Cancer Genome Consortium
RT-PCR: Reverse transcription-polymerase chain reaction
PPI: Protein-protein interaction
miRNA: MicroRNA
lncRNA: Long-chain noncoding RNA.

## Supplementary Materials

Supplementary Materials Table 1: the expression of m6A genes in TCGA. Table 2: the clinical information of patients in TCGA. Table 3: clinicopathological characteristics of patient samples. Table 4: primer sequence.
