# Supplementary material for: m6A-Related Genes Contribute to Poor Prognosis of Hepatocellular Carcinoma
Source: Comput Math Methods Med. 2022 Oct 26;2022:2427987. doi: 10.1155/2022/2427987 (PMC9629938; doi:10.1155/2022/2427987)
Supplement: Supplementary Materials — Table 1: the expression of m6A genes in TCGA. Table 2: the clinical information of patients in TCGA. Table 3: clinicopathological characteristics of patient samples. Table 4: primer sequence. [file 2427987.f1.zip › clinical data only for review.pdf]

NO	Age	Gender	AFP	HBsAg	Cirrhosis	TumorSize	Tumournu	Vascularin	Capsule	Distantmet	TNMstage	DFS	OS
1	1	2	2	1	2	1	1	1	2	2	3	8	8
2	2	2	1	1	2	1	2	1	2	1	3	5	9
3	1	2	1	1	2	1	2	1	2	1	3	6	10
4	2	2	1	2	1	1	2	1	1	1	3	6	10
5	2	1	1	2	1	1	2	1	2	1	3	6	11
6	1	1	1	1	1	2	1	1	2	1	3	6	12
7	1	1	1	1	1	1	2	1	2	1	3	7	12
8	2	2	1	2	2	2	2	2	2	1	3	8	12
9	2	1	1	2	1	1	2	2	2	1	3	8	13
10	1	1	1	1	1	1	2	1	2	1	3	7	13
11	1	2	1	1	2	1	2	1	1	1	3	9	13
12	2	1	2	1	1	2	2	1	1	1	3	7	14
13	2	1	2	2	1	1	2	1	1	1	3	10	15
14	1	2	1	1	2	1	1	2	1	1	3	9	15
15	1	2	2	1	1	2	2	2	2	1	3	9	15
16	1	2	2	1	2	2	2	1	2	1	3	9	16
17	2	1	2	2	1	1	1	2	1	1	3	9	18
18	1	1	2	1	1	2	1	1	2	1	3	12	19
19	2	1	2	2	1	1	2	2	2	1	3	14	21
20	1	1	2	1	1	2	1	1	2	1	3	15	22
21	2	1	1	2	1	1	1	1	1	2	3	16	23
22	1	2	1	1	2	1	1	1	2	2	3	17	28
23	1	1	2	1	1	1	1	1	2	2	3	17	29
24	1	1	2	1	1	2	1	2	1	2	3	21	29
25	1	2	2	1	2	2	2	2	2	2	3	21	29
26	2	1	1	2	1	1	1	2	2	2	2	19	30
27	1	1	2	1	1	1	1	1	1	2	3	22	30
28	2	1	2	2	1	2	2	2	2	2	2	22	30
29	1	2	2	1	2	2	1	2	2	2	3	22	30
30	1	2	2	1	2	1	2	2	1	2	3	21	31
31	2	2	2	2	2	1	2	2	2	2	3	22	31

32	1	1	2	1	1	1	2	2	2	2	2	22	31
33	2	2	2	1	2	2	2	1	2	2	3	23	31
34	2	1	2	2	1	1	1	2	2	2	3	23	32
35	1	1	2	1	1	2	1	2	2	2	3	24	33
36	1	1	2	1	2	1	1	2	2	2	3	19	33
37	1	1	2	1	2	1	2	1	2	2	3	23	33
38	2	1	2	2	1	2	2	2	2	1	3	25	33
39	1	1	2	1	2	1	2	2	2	1	2	29	39
40	1	1	1	1	1	2	1	1	1	1	2	34	43
41	1	2	2	1	2	2	2	1	1	1	3	31	44
42	1	1	1	1	1	1	1	2	2	1	2	37	45
43	1	1	2	1	1	2	1	1	1	1	3	33	45
44	1	1	2	1	1	2	1	1	2	1	3	33	45
45	1	2	2	1	2	2	1	2	2	1	2	33	45
46	2	1	2	2	1	1	2	2	1	1	3	31	46
47	2	1	2	1	1	2	2	1	2	1	3	33	46
48	2	2	2	2	2	2	1	2	2	1	2	33	46
49	1	2	2	1	2	2	1	1	2	1	3	34	46
50	1	2	2	1	2	1	2	1	2	1	2	35	47
51	2	2	2	2	2	2	2	2	1	1	3	34	48
52	2	2	2	2	2	2	2	2	1	1	3	34	48
53	1	1	1	1	1	1	2	1	1	2	2	37	48
54	1	1	1	1	1	1	2	1	1	2	2	37	48
55	2	2	1	2	2	2	1	2	2	2	2	33	49
56	2	2	1	2	2	2	1	2	2	2	3	33	49
57	1	1	1	1	1	2	1	2	2	2	3	36	49
58	1	1	1	1	1	2	1	2	2	2	3	36	49
59	1	1	1	1	1	1	1	1	1	2	2	35	50
60	1	1	1	1	1	1	1	1	1	2	2	35	50
61	1	2	1	1	2	1	1	2	2	2	2	57	57
62	1	2	1	1	2	1	1	2	2	2	2	57	57
63	1	1	2	1	1	1	1	2	2	1	2	57	57

64	1	1	2	1	1	1	1	2	2	1	2	57	57
65	1	1	2	1	1	1	1	2	1	2	3	44	58
66	1	1	2	1	1	1	1	2	1	2	3	44	58
67	2	1	1	2	1	1	2	1	1	2	3	63	63
68	2	1	1	2	1	1	2	1	1	2	3	63	63
69	1	2	2	1	2	2	1	2	1	2	3	63	63
70	1	2	2	1	2	2	1	2	1	2	3	63	63
71	1	1	1	1	1	2	1	1	1	2	3	51	65
72	1	1	1	1	1	2	1	1	1	2	3	51	65
73	1	2	1	1	2	2	2	2	2	2	3	66	66
74	1	2	1	1	2	2	2	2	2	2	3	66	66
75	1	2	1	1	2	1	2	2	2	1	2	55	67
76	1	2	1	1	2	1	2	2	2	1	2	55	67
77	2	2	1	2	2	2	2	2	1	2	3	67	67
78	2	2	1	2	2	2	2	2	1	2	3	67	67
79	1	2	1	1	2	1	1	2	2	2	2	67	67
80	1	2	1	1	2	1	1	2	2	2	2	67	67
81	2	2	1	1	2	2	2	2	1	2	1	68	68
82	2	2	1	1	2	2	2	2	1	2	2	68	68
83	2	1	2	2	1	2	2	2	1	2	3	68	68
84	2	1	2	2	1	2	2	2	1	2	3	68	68
85	2	1	1	1	1	1	2	2	1	2	1	68	68
86	2	1	1	1	1	1	2	2	1	2	3	68	68
87	2	1	2	1	1	2	2	2	2	2	3	69	69
88	2	1	2	1	1	2	2	2	2	2	3	69	69
89	2	2	1	2	2	1	2	2	1	2	2	65	72
90	2	2	1	2	2	1	2	2	1	2	2	65	72
91	2	1	1	2	1	1	2	2	1	2	1	73	73
92	1	2	2	1	2	2	2	1	1	2	3	77	77
93	1	2	2	1	2	2	2	2	1	2	1	77	77
94	2	1	1	2	1	1	2	2	2	2	2	78	78
95	2	1	1	2	1	2	2	1	1	1	1	78	78

96	1	2	2	1	2	2	2	2	1	2	1	80	80
97	2	2	2	2	2	2	2	1	1	2	1	80	80
98	1	1	2	1	1	2	2	1	2	2	1	81	81
99	1	2	2	1	2	2	2	2	1	2	1	85	85
100	1	2	2	1	2	2	2	2	1	2	1	85	85
101	2	1	1	2	1	2	2	2	1	2	1	90	90
102	2	2	1	2	2	1	1	2	2	1	2	9	17
103	2	1	1	2	1	2	1	1	2	2	3	9	18
104	1	1	1	1	1	1	1	1	2	1	2	10	18
105	1	1	1	1	1	1	1	2	2	1	2	10	18
106	2	2	1	2	2	1	1	2	2	1	2	12	18
107	1	1	1	1	1	1	1	2	2	1	2	11	20
108	2	2	1	2	2	2	2	2	2	1	2	13	20
109	1	2	2	1	2	1	1	1	2	1	2	24	24
110	2	1	1	2	1	1	1	2	2	1	1	18	29
Explain	< 65, 2<br>⩾ 65, 1	male, 1<br>female, 2	< 20, 1<br>⩾ 20, 2	1<br>Negative,	Present, 1<br>Absent, 2	< 5cm, 2<br>⩾ 5cm, 1	1<br>Solitary, 2	Yes, 1<br>No, 2	Present, 2<br>Absent, 1	Present, 2<br>Absent, 1	T1, 1; T2, 2;<br>T3, 3; T4, 4	month	month

END	YTHDF2	Adjacent	YTHDF1	Adjacent	METTL3	Adjacent	KIAA1429	Adjacent	Risk score
0	4.38944	1.46775	2.350351	0.820281	2.8096	0.556853	2.388226	1.413843	0.58907
0	2.173673	1.428597	1.015543	0.124347	8.308	0.896675	1.977292	0.805073	0.771222
0	5.25712	0.69525	3.887049	1.149639	7.6379	0.329863	2.522086	0.922839	1.004973
0	2.180338	0.944341	1.038908	0.485572	0.4017	0.656739	1.851162	1.22558	0.246142
0	4.35	0.78	2.285964	0.768771	9.885	0.421847	2.99883	1.379272	1.072741
0	1.898208	0.622217	0.778775	0.222207	2.9433	0.192245	2.551006	1.327478	0.414013
0	5.26988	0.296125	3.986945	1.229556	9.781	0.465307	2.047209	0.62384	1.133722
0	1.909176	1.098062	0.839472	0.41882	0.3751	0.195653	3.323	1.323	0.273046
0	1.915291	0.657301	0.847365	0.130296	2.9433	0.383104	2.067023	1.004096	0.400418
0	0.5742	0.141625	0.516364	0.446788	8.181	0.116315	3.8283	1.8283	0.717928
0	5.26988	0.296125	3.998505	1.238804	7.6884	0.147713	2.972134	1.301676	1.02737
0	2.191449	0.569254	1.04421	0.488678	3.2901	0.520666	2.460983	0.889037	0.458309
0	2.234301	0.617447	1.058067	0.191358	8.586	0.498678	2.024044	0.785413	0.795885
0	2.196826	1.090728	1.053869	0.3168	8.512	0.066313	1.849155	0.720992	0.78273
0	4.38944	0.43775	2.352318	0.821854	0.6779	0.715477	2.173565	1.205958	0.441132
0	4.38944	1.46775	2.355165	0.824132	0.6779	0.367065	3.3483	1.3483	0.48115
0	1.25048	0.399125	0.600017	0.427353	0.1183	0.652673	2.114399	1.285802	0.169676
0	2.237716	0.655472	1.061491	0.442945	0.4017	0.989868	3.018506	1.489848	0.289827
0	4.4022	0.270375	3.207496	0.605997	0.7266	0.977414	3.4167	1.4167	0.510456
0	1.30085	0.665232	0.6201	0.205626	2.8396	0.216134	2.402018	1.472485	0.362575
0	4.4022	0.579375	3.208075	0.60646	5.9447	0.32037	1.926323	0.968638	0.804193
0	5.3592	0.43775	4.017974	1.254379	0.8051	0.097973	2.173673	1.428597	0.55172
0	2.27128	1.866875	1.076307	0.120205	3.2901	0.040301	2.915693	1.178106	0.479346
0	1.926323	0.968638	0.84837	0.29038	0.4287	0.961953	2.030894	0.614904	0.233904
0	4.77224	0.9785	3.286153	0.668922	6.4287	0.383104	2.557162	0.846417	0.881526
0	1.42912	2.587875	0.642416	0.171219	0.964	0.425214	3.036593	1.343982	0.268531
0	2.279798	0.848673	1.133121	0.571575	0.5351	0.454716	2.572297	0.703241	0.287877
0	1.977292	0.805073	0.858917	0.206998	2.4068	0.550454	2.240849	0.922886	0.374889
0	1.535422	1.171105	0.648893	0.3132	0.9644	0.141046	2.58265	1.139139	0.259571
0	2.27128	1.866875	1.131384	0.225762	2.4188	0.055424	2.279798	0.848673	0.401707
0	1.643006	0.635962	0.66257	0.316393	2.3651	0.040301	1.909176	1.098062	0.335835

0	2.283063	1.346344	1.17609	0.435104	1.1968	0.576471	2.234301	0.617447	0.32141
0	2.323044	1.04877	1.182925	0.180737	1.1968	0.885254	2.792787	0.923446	0.342942
0	2.35856	1.068356	1.202308	0.462213	1.323	0.825196	2.828326	1.465964	0.355098
0	2.388226	1.413843	1.224019	0.27501	1.3362	0.967428	1.772545	0.725684	0.32241
0	0.66352	1.403375	0.518586	0.548496	2.8396	0.667693	2.391197	0.612142	0.321864
0	2.024044	0.785413	0.862732	0.242299	1.0889	0.47837	1.898208	0.622217	0.279119
0	2.402018	1.472485	1.237834	0.172703	2.4188	0.732059	2.283063	1.346344	0.412406
0	2.414775	0.899967	1.248275	0.491365	2.5392	0.572581	2.323044	1.04877	0.422746
0	4.77224	0.9785	3.31459	0.691672	1.8283	0.861466	3.1709	1.1709	0.599534
0	2.029236	1.225808	0.888189	0.131615	1.0889	0.306488	2.77739	1.16013	0.310005
0	2.067023	1.004096	0.97048	0.173689	3.0806	0.352365	2.460777	0.746798	0.435143
0	2.030894	0.614904	0.892015	0.481933	7.0283	0.426329	2.942156	1.040024	0.707808
0	5.49956	0.527875	4.140478	1.352382	6.829	0.683209	2.510151	1.222467	0.972326
0	2.460983	0.889037	1.267417	0.474242	1.4167	0.859581	2.237716	0.655472	0.349003
0	2.460777	0.746798	1.252524	0.411375	7.3545	0.045833	2.959077	1.068288	0.76501
0	1.772545	0.725684	0.672065	0.114261	1.0804	0.12559	2.757638	1.081572	0.287792
0	1.832948	1.153923	0.692194	0.440229	2.9251	0.32037	2.414775	0.899967	0.401992
0	2.48904	2.001361	1.272556	0.234797	3.4895	0.941226	1.915291	0.657301	0.476639
0	2.040935	0.723622	0.927031	0.419078	1.1302	0.957054	3.0804	1.0804	0.324772
0	2.510151	1.222467	1.285768	0.491728	3.6268	0.550454	3.1968	1.1968	0.530875
0	2.507291	0.928939	1.277949	0.249642	3.6268	0.055424	1.923609	0.846688	0.487206
0	1.151188	1.464845	0.571482	0.4061	0.8978	0.126194	2.180338	0.944341	0.216736
0	0.66352	1.403375	0.567408	0.420203	0.8543	0.114582	1.182476	1.388896	0.151056
0	5.66544	1.429125	4.295176	1.476141	2.0981	0.382239	2.597969	1.188371	0.677036
0	5.49956	0.527875	4.277175	1.46174	2.071	0.62791	2.895718	0.630579	0.675098
0	2.047209	0.62384	0.952862	0.542663	0.4921	0.493703	2.735983	0.984887	0.272015
0	2.04336	0.930773	0.927808	0.307937	0.4921	0.453252	2.040935	0.723622	0.247479
0	5.24436	0.6695	3.581964	0.905571	1.8367	0.551153	2.85341	1.196566	0.624368
0	5.07848	0.43775	3.495186	0.836149	1.8346	0.211025	2.849391	1.429718	0.611963
0	2.536486	1.06667	1.321232	0.297506	1.4514	0.566055	1.836309	1.201722	0.343553
0	2.533332	1.49567	1.312161	0.540369	1.4514	0.533221	1.832948	1.153923	0.343008
0	5.25712	1.120125	3.686978	0.989582	1.9892	0.352365	2.890352	0.829459	0.639278

0	5.24436	0.6695	3.638241	0.950593	6.4919	0.192245	1.30085	0.665232	0.880344
0	2.522086	0.922839	1.294825	0.454633	4.043	0.572581	2.934537	1.089249	0.550376
0	2.520118	0.589128	1.286698	0.19492	3.6295	0.732059	1.839413	1.432249	0.485515
0	1.839413	1.432249	0.730471	0.188518	0.2065	0.686088	2.721299	0.54962	0.234401
0	1.836309	1.201722	0.715808	0.249706	0.1962	0.163939	2.713867	0.601271	0.23289
0	2.557162	0.846417	1.339321	0.287899	4.5561	0.056983	2.676352	1.067054	0.578733
0	2.551006	1.327478	1.336928	0.441842	4.4549	0.052855	2.48904	2.001361	0.565257
0	1.182476	1.388896	0.587027	0.223391	0.94	0.203512	2.191449	0.569254	0.222165
0	1.15502	0.908736	0.576429	0.489821	0.8978	0.564131	3.47	1.352252	0.260945
0	2.572297	0.703241	1.407702	0.22817	5.2238	0.556853	2.67682	1.082176	0.625556
0	2.558297	1.059773	1.395657	0.47066	4.9521	0.598126	1.151188	1.464845	0.554601
0	2.114399	1.285802	0.975797	0.331894	3.2236	0.382239	2.095384	0.638567	0.435097
0	2.095384	0.638567	0.970619	0.277722	3.0913	0.62791	2.914935	1.303579	0.452968
0	2.592684	0.573892	1.428134	0.177533	9.025	0.661538	2.533332	1.49567	0.873311
0	2.58265	1.139139	1.423516	0.42455	8.586	0.416038	2.029236	1.225808	0.826481
0	7.79636	0.424875	4.321476	1.497181	2.3651	0.941226	2.899443	0.820139	0.831343
0	6.53312	0.296125	4.309321	1.487457	2.3459	0.520666	2.671655	0.834903	0.747471
0	3.13	2.1	1.473069	0.314456	2.6098	0.056983	2.904331	1.363544	0.495437
0	2.597969	1.188371	1.429457	0.211451	2.5723	0.052855	2.35856	1.068356	0.441838
0	3.68764	1.454875	1.491944	0.324155	5.6197	0.667693	3.25	0.917638	0.739253
0	2.6796	1.583625	1.439162	0.450445	5.3685	0.216134	3.1968	1.1968	0.659966
0	3.68764	1.454875	1.50012	0.111848	1.5563	0.270413	2.848208	0.867361	0.457629
0	2.71788	0.270375	1.458599	0.129542	1.5296	0.487629	3.56	0.639561	0.421731
0	3.98112	1.300375	1.51398	0.228147	7.4666	0.51646	2.520118	0.589128	0.854243
0	3.96836	0.1545	1.505566	0.24541	7.4231	0.466099	2.967865	1.011579	0.865616
0	2.173565	1.205958	1.000701	0.291265	1.1746	0.207116	2.196826	1.090728	0.307475
0	2.15256	1.221591	0.986786	0.242825	1.1709	0.966988	3.0804	1.0804	0.335657
0	7.79636	0.424875	4.343429	1.514743	10	0.681536	1.15502	0.908736	1.276529
0	1.849155	0.720992	0.742013	0.118865	2.9251	0.013322	2.536486	1.06667	0.408432
0	3.98112	0.193125	1.518651	0.113938	0.55	0.22928	2.558297	1.059773	0.399172
0	5.25712	3.99125	3.775253	1.060202	6.6159	0.377833	2.507291	0.928939	0.933999
0	3.98112	1.300375	1.531384	0.229713	9.645	0.917184	2.04336	0.930773	0.982278

0	3.98112	0.193125	1.53552	0.439053	1.6081	0.447064	1.535422	1.171105	0.434684
0	5.25712	0.69525	3.776988	1.06159	1.9236	0.262026	2.878498	1.317079	0.636975
0	4.10872	1.00425	2.101758	0.621406	5.8122	0.013322	2.937864	1.071926	0.782655
0	4.26184	0.84975	2.190774	0.692619	2.7851	0.598126	2.905999	1.023213	0.59322
0	5.25712	2.99125	3.877639	1.142111	0.0976	0.261414	1.643006	0.635962	0.47717
0	1.851162	1.22558	0.749223	0.483659	0.2821	0.054201	2.722711	1.465178	0.240638
1	1.25048	0.399125	0.600852	0.275296	0.1522	0.727437	2.712034	1.279272	0.192256
1	4.38944	0.43775	2.541793	0.973434	1.6484	0.545586	2.592684	0.573892	0.524551
1	4.4022	0.270375	3.008799	1.347039	7.4666	0.462532	2.98356	1.199571	0.935204
1	1.923609	0.846688	0.847586	0.518578	1.0804	0.542217	2.760302	0.633099	0.301534
1	2.240849	0.922886	1.068353	0.259602	1.1746	0.834207	2.2	1.247923	0.313379
1	4.4022	0.579375	3.189423	0.591538	9.941	0.220973	3.000062	0.836242	1.103952
1	1.276	2.987	0.612866	0.174926	0.4287	0.181178	2.15256	1.221591	0.193313
1	2.391197	0.612142	1.228075	0.327563	1.3483	0.39488	3.5563	1.5563	0.384141
1	1.36	1.34	0.631794	0.110175	0.964	0.264133	2.750037	0.970773	0.254424

dead, 0
live, 1

Cutoff value=0.4552984155
