# Supplementary material for: m6A-Related Genes Contribute to Poor Prognosis of Hepatocellular Carcinoma
Source: Comput Math Methods Med. 2022 Oct 26;2022:2427987. doi: 10.1155/2022/2427987 (PMC9629938; doi:10.1155/2022/2427987)
Supplement: Supplementary Materials — Table 1: the expression of m6A genes in TCGA. Table 2: the clinical information of patients in TCGA. Table 3: clinicopathological characteristics of patient samples. Table 4: primer sequence. [file 2427987.f1.zip › Sup Tab 3 (1).docx]

**Supplementary Table 3. Clinicopathological characteristics of patient samples**

| **Characteristics** | **N (%)** |
| --- | --- |
|  |  |
| **Age** |  |
| ≥65 | 63 (57.3%) |
| <65 | 47 (42.7%) |
| **Gender** |  |
| Male | 60 (54.5%) |
| Female | 50 (45.5%) |
| **AFP μg/l** |  |
| <20 | 56 (50.9%) |
| ≥20 | 54 (49.1%) |
| **HbsAg** |  |
| Positive | 73 (66.4%) |
| Negative | 37 (33.6%) |
| **Cirrhosis** |  |
| Present | 59 (53.6%) |
| Absent | 51 (46.4%) |
| **Tumor size** |  |
| ≥5cm | 57 (51.8%) |
| <5cm | 53 (48.2%) |
| **Tumor number** |  |
| Mulitple | 49 (44.5%) |
| Solitary | 61 (55.5%) |
| **Vascular invasion** |  |
| Yes | 42 (38.2%) |
| No | 68 (61.8%) |
| **Capsule** |  |
| Absence | 47 (42.7%) |
| Presence | 63 (57.3%) |
| **Distant metastasis** |  |
| Absence | 47 (42.7%) |
| Presence | 63 (57.3%) |
| **TNM stage** |  |
| I | 12 (10.9%) |
| II | 33 (30.0%) |
| III | 65 (59.1%) |
