# Supplementary material for: m6A-Related Genes Contribute to Poor Prognosis of Hepatocellular Carcinoma
Source: Comput Math Methods Med. 2022 Oct 26;2022:2427987. doi: 10.1155/2022/2427987 (PMC9629938; doi:10.1155/2022/2427987)
Supplement: Supplementary Materials — Table 1: the expression of m6A genes in TCGA. Table 2: the clinical information of patients in TCGA. Table 3: clinicopathological characteristics of patient samples. Table 4: primer sequence. [file 2427987.f1.zip › Sup Table 4 (1).docx]

|  | F 5-3 | R 5-3 |
| --- | --- | --- |
| YTHDF2 | ACAAAAGCCTCCGCCTGCT | GACCTTTTGGTCTCTGCTCCAA |
| YTHDF1 | CGTGGACACCCAGAGAACAA | CGCTCATTGAGGGGTAACTGT |
| METTL3 | TTTTCCGGTTAGCCTTCGGG | GATAGAGCTCCACGTGTCCG |
| KIAA1429 | ATTTTCCGGTTAGCCTTCGGG | TCCACGTGTCCGACATCCTA |

Supplement Table 4. Primerb Sequence
